# Family costs for pediatric hospitalized respiratory syncytial virus lower respiratory tract infections: an Italian multicenter study

**DOI:** 10.1186/s13052-025-02154-w

**Published:** 2025-12-02

**Authors:** Elena Bozzola, Enza D’Auria, Anna Chiara Vittucci, Sandra Trapani, Diego Peroni, Susanna Esposito, Raffaele Badolato, Antonietta Giannattasio, Emanuela Piccotti, Eugenio Baraldi, Andrea Marcellusi

**Affiliations:** 1 Pediatric Unit, Bambino Gesù Children’s Hospital, IRCCS, Rome, Italy; 2 Department of Pediatrics, Buzzi Children’s Hospital, University of Milan, Milan, Italy; 3 Department of Biomedical and Clinical Sciences, University of Milan, Milan, Italy; 4 Department of Health Sciences, University of Florence, Florence, Italy; 5 Meyer Children’s Hospital IRCCS, Paediatric Unit, Florence, Italy; 6 UO Paediatric Clinic, Azienda Ospedaliera-Universitaria di Pisa, Pisa, Italy; 7 Pediatric Clinic, University Hospital of Parma, Parma, Italy; 8 Department of Paediatrics of the “Spedali Civili di Brescia”, Brescia, Italy; 9 Pediatric Emergency Department, Santobono-Pausilipon Children’s Hospital, Naples, Italy; 10 Paediatric Emergency Medicine, Istituto Giannina Gaslini, IRCCS, Genova, Italy; 11 Dipartimento Salute Donna e Bambino, Azienda Ospedale-University of Padova, Padova, Italy; 12 Department of Pharmaceutical Science (DISFARM), University of Milan, Milan, Italy

**Keywords:** Respiratory syncytial virus, Lower respiratory tract disease, Children, Hospitalization, Cost

## Abstract

**Background:**

Respiratory syncytial virus (RSV) is a leading cause of severe lower respiratory tract disease, particularly in young children. It represents a significant cause of morbidity and mortality worldwide, as well as a substantial cost driver in cases requiring hospitalization. The aim of the study was to generate data on the direct and indirect costs borne by families of children hospitalized with RSV-associated acute lower respiratory tract infection (ALRTI).

**Material and methods:**

Inpatient children aged 24 months of age or below affected by ALRTI were enrolled at nine Italian pediatric hospitals. The study was conducted between November and March of 2022–2024, covering two consecutive RSV seasons in Italy. Direct and indirect costs incurred by families were collected through questionnaires completed by the parents at admission (T0) and again after 7 days (T1).

**Results:**

A total of 296 patients were enrolled during the study period and divided into two groups according to nasal swab results: 225 RSV-positive (76%) and 71 RSV-negative (24%). Family finances were reported to be affected by direct costs in 79% of the RSV+ group and 63.1% of the RSV-group. Direct costs were similar at T0 (€ 52.5 ± 94.6 in RSV+ group and € 53.5 ± 171.4 in RSV- group), but higher at T1 in the RSV+ group (€ 53.8 ± 126.6 vs € 32.3 ± 64.4). Regarding indirect costs, a greater proportion of parents in the RSV-positive group reported taking additional paid leave from work at T1 (41.8% vs 25.3%; p-value = 0.0426) as well as more unpaid leave (13.7% vs 7%).

**Discussion:**

This study highlights the substantial economic burden of RSV-related hospitalizations from the household perspective, encompassing both direct out-of-pocket expenses and indirect costs due to work absenteeism across two consecutive epidemic seasons. By documenting the real-life financial impact of RSV on families, our findings provide an important parent-centered perspective, support a more comprehensive evaluation of the RSV burden and underscore the need for broad preventive measures against the disease.

## Background

Acute lower respiratory tract infection (ALRTI) due to respiratory syncytial virus (RSV) is a main cause of hospitalizations and one of the most common causes of preventable pediatric deaths in the world [1]. Infection with RSV is seen worldwide in annual epidemics which in Italy usually last 5 months occurring between October and March, with a peak between December and February [2]. More than 90% of children are infected by the age of two years and re-infection is common [2]. RSV accounts for 40% of hospital admissions for pneumonia and up to 80% of hospital admissions for bronchiolitis in infants aged < 1 year, while rates of illness are even higher among infants ≤6 months of age [3, 4].

Considering the overall economic burden of illness, RSV accounts for 1.2 million discounted disability-adjusted life-years and is associated with a financial toll of approximately €4.82 billion per year globally, considering both inpatients and outpatients under 5 years of age [5]. However, Economic studies are still scarce, and most of them focus on assessing the acute hospitalization costs, rather than the social perspective, and so do not include indirect costs, related to lost workdays, reduced income, and the need to reorganize household and professional responsibilities.

There are three RSV prevention products approved for use in Italy: RSVpreF maternal vaccine, the long-acting monoclonal antibody (mAb) named nirsevimab, and the short acting mAb called palivizumab. While nirsevimab is recommended in all infants during their first RSV season and is being widely used across Italy during the season 2024/2025, the adoption of the RSVpreF maternal vaccine remains scarce in the same period; instead, palivizumab is now only recommended in case of nirsevimab shortage. Finally, many other vaccines and immunotherapeutic technologies are under clinical development and evaluation for children [6–9].

The aim of the study is to generate data on the direct and indirect costs of taking care of a child hospitalized with RSV-associated ALRTI from the household’s perspective.

## Methods

RESPIRIAMO is a not-for-profit, prospective, multi-center, epidemiological study conducted from 2022 to 2024 in children aged 0 to 24 months who were hospitalized for RSV ALRTI in Italy. The study has been sponsored by Italian Network for Paediatric Clinical Trials (INCIPIT), a not-for-profit Consortium composed of the main Italian pediatric hospitals and Scientific Institutes for Research, Hospitalization and Healthcare and various pediatric Research Units general hospitals. The study was designed to represent the Italian population of children under two years through a multi-center sample, including cities from the Northern, Central, and Southern Italy.

Inpatient children aged 24 months of age or below affected by ALRTI were enrolled in the study at nine Italian pediatric hospitals, belonging to the INCIPIT network. The study period ran between November and March of 2022 to 2024 (i.e. two consecutive RSV seasons in Italy). All readmissions following discharge were excluded.

ALRTI was defined as the presence of at least one manifestation of lower respiratory tract infection signs (cough, nasal flaring, indrawing of the lower chest wall, subcostal retractions, stridor, rales, rhonchi, wheezing, crackles or crepitations, or observed apnea) plus hypoxemia (peripheral oxygen saturation of < 95% on room air) and/or tachypnea (≥70 breaths per minute from 0 to 59 days of age, and ≥60 breaths per minute at 60 days of age or older) [10–12]. A panel of respiratory pathogens, including RSV, was identified through quantitative real-time polymerase chain reaction by the research laboratory, independently, on the basis of the hospital’s diagnostic method [10].

The data for this analysis were derived from the structured case report forms completed by the study physicians and from 2 questionnaires administered to parents at hospital admission (T0) and 7 days after (T1). Questionnaires were completed at enrolment by caregivers (mother, father or legal tutor) to record infants’ and caregivers’ background characteristics (including socio-demographics and potential risk factors). The number of days absent from work and family-related costs, including over-the-counter medications, homemade remedies, public and private transport, and supplemental child assistance, were extracted from the questionnaires. Parents estimated their expenses for medication, and supplemental childcare due to ALRTI of their child. To determine the travel expenses of the parents, the calculation was based on the reimbursed costs per km by the statutory sickness funds (€0.22 per km). All direct medical costs were evaluated from the families’ objective perspective, and no hospital costs have been added. Indirect costs associated with RSV, represented by absenteeism from work as paid or unpaid vacations, were assessed. Calculated from the data on gross income and number of employees in the 2024 Statistical Yearbook, the monetary value of productivity for employees was € 76.5 per day. [13]

A nasal swab was obtained from a depth of 2–3 cm in the nostril using of a sterile cotton swab which was then inserted into a vial containing a viral transport medium. The detection of RSV in the specimens was based on both viral culture and reverse-transcription polymerase chain reaction. Based on the results, patients were divided into two groups: RSV-positive patients and RSV-negative patients.

RESPIRIAMO study has been conducted in accordance with the Good Clinical Practice (with its general principles, although its observational nature) in order to ensure that the data collected are in compliance with the ALCOAC standards for Source Data and Source Documents (Attributable, Legible, Contemporaneous, Original, Accurate, and Complete).

Study data were reported in a validated eCRF and checked for completeness and consistency by an appointed data manager and during site visits by the appointed Clinical Research Associates identified by the Sponsor. The database was locked once all the monitoring checks were completed.

## Ethical considerations

The institutional review boards at each participating hospital approved the study. Informed consent was obtained from all parents or legal guardians of the subjects participating into the study.

## Statistical analysis

Continuous variables, such as the age, are presented as mean ± SD, the comparisons between the RSV Positive/Negative groups were carried out with the Wilcoxon-Mann-Whitney test. Categorical variables, such as breastfeeding, are presented as numbers and percentages, the comparisons between the RSV Positive/Negative groups were carried out with the chi-square (or Fisher) test. All statistical tests were two-tailed, and the significance level used was *p* < 0.05.

## Results

According to the inclusion criteria, 296 patients were prospectively enrolled during two consecutive respiratory illness seasons. On the base of the nasal swab findings, they were divided into two groups, of which 225 RSV-positive (76%) and 71 RSV-negative (24%). Of note, a higher percentage of RSV- patients were premature and older than RSV+ patients. The characteristics of the patients are presented in Table 1.

**Table 1:** Sociodemographic and clinical characteristics of patients

Parameters	Statistics	RSV Positive group N = 225	RSV Negative group N = 71	p-value
Age (month)	Mean ± SD (min-max)	4.4 ± 4.2 (0.5–22)	6.4 ± 5.7 (0.5–21)	0.0373 *
Gender M/F	N (%)	119 (52.9%)/106 (47.1%)	41 (57.8%)/30 (42.3%)	0.4739
Sibling < 5 years of age 5–15 years of age > 15 years of age	Mean ± SD Mean ± SD Mean ± SD	0.86 ± 0.6 0.42 ± 0.7 0.07 ± 0.3	0.84 ± 0.6 0.5 ± 0.6 0.05 ± 0.2	0.9211 0.1982 0.8331
Premature birth	N (%)	22 (10.0%)	14 (20.0%)	0.0271 *
Breastfeeding	N (%)	171 (82.6%)	54 (78.3%)	0.4203
Family history of atopia, eczema, asthma	N (%)	31 (16.0%)	13 (19.7%)	0.4203
Oxygen saturation at admission (%)	Mean ± SD	94.68 ± 4.0	95.17 ± 3.7	0.3791
Respiratory rate at admission (rate/min)	Mean ± SD	53.53 ± 13.6	53.05 ± 12.5	0.7620
Temperature at admission (°C)	Mean ± SD	37.03 ± 0.9	37.22 ± 1.1	0.3399

^*^Significant value; SD: standard deviation

In a sensitivity analysis restricted to RSV-positive patients, we further explored the potential effect of prematurity by stratifying the cohort into preterm and non-preterm infants. This approach allowed us to assess whether prematurity may have influenced the observed family costs (Table 2).

**Table 2:** RSV premature and not premature patients

Parameters	Statistics	Premature N = 22	Not premature N = 203	p-value
Age (month)	Mean ± SD (min-max)	4.9 ± 5.0 (1–21)	4.4 ± 4.1 (0.5–22)	0.6010
Gender F/M	N (%)	9 (40.9%)/13 (59.1%)	97 (47.8%)/106 (52.2%)	0.5395
Sibling < 5 years of age 5–15 years of age > 15 years of age	Mean ± SD Mean ± SD Mean ± SD	1.0 ± 0.73 0.45 ± 0.69 0.25 ± 0.46	0.84 ± 0.62 0.42 ± 0.68 0.05 ± 0.26	0.3481 0.8557 0.0555
Breastfeeding	N (%)	12 (54.5%)	159 (78.3%)	0.0187*
Family history of atopia, eczema, asthma	N (%)	3 (13.6%)	28 (13.8%)	1
Oxygen saturation at admission (%)	Mean ± SD	93.5 ± 3.8	94.8 ± 4.0	0.1434
Respiratory rate at admission (rate/min)	Mean ± SD	54.7 ± 13.5	53.4 ± 13.6	0.6968
Temperature at admission (°C)	Mean ± SD	37.1 ± 0.9	37.0 ± 0.9	0.9779

^*^Significant value: SD: standard deviation

Almost all patients declared not to have a private health insurance but only the public healthcare assistance (99,3% RSV+ group; 100% RSV- group).

Family finances were reported to have been affected in 79% of the RSV+ group and 63.1% of the RSV-group by direct costs. In detail, in the first questionnaire (T0), 70.7% of the parents of the RSV+ group referred spending money on over-the-counter medications - € 34.9 ± 38.7), homemade remedies (€ 26.0 ± 19.3), car and other means of transport (€ 40.1 ± 75.6 and € 53.3 ± 56.1), supplemental child assistance (€ 175.7 ± 276.8) or other expenses (€ 61.1 ± 46.1). As for RSV- group, 55.2% of parents had to pay as well for over-the-counter medications (€ 37.0 ± 50.5), homemade remedies (€ 60.0), car and other means of transport (€ 53.6 ± 56.3 and 26.8 ± 28.1 or others (€ 96.3 ± 114.4). After 7 days following the admission, at the second questionnaire (T1), 49.3% of parents of RSV+ group spent additional money for over-the-counter medications (€ 20.9 ± 13.2), homemade remedies (€ 20.0 ± 28.3), car and other means of transport (€ 96.2 ± 150.5 and 66.7 ± 75.9), supplemental child assistance (€ 70.0 ± 55.2) or others (€ 73.3 ± 117.1). As for RSV- group, only 22.3% of parents had to pay for over-the-counter medications (€ 33.3 ± 25.2), car and other means of transport (€ 60.2 ± 75.0 and 26.7 ± 5.8 or other expenses (€ 98.0 ± 88.2).

**Fig. 1:**
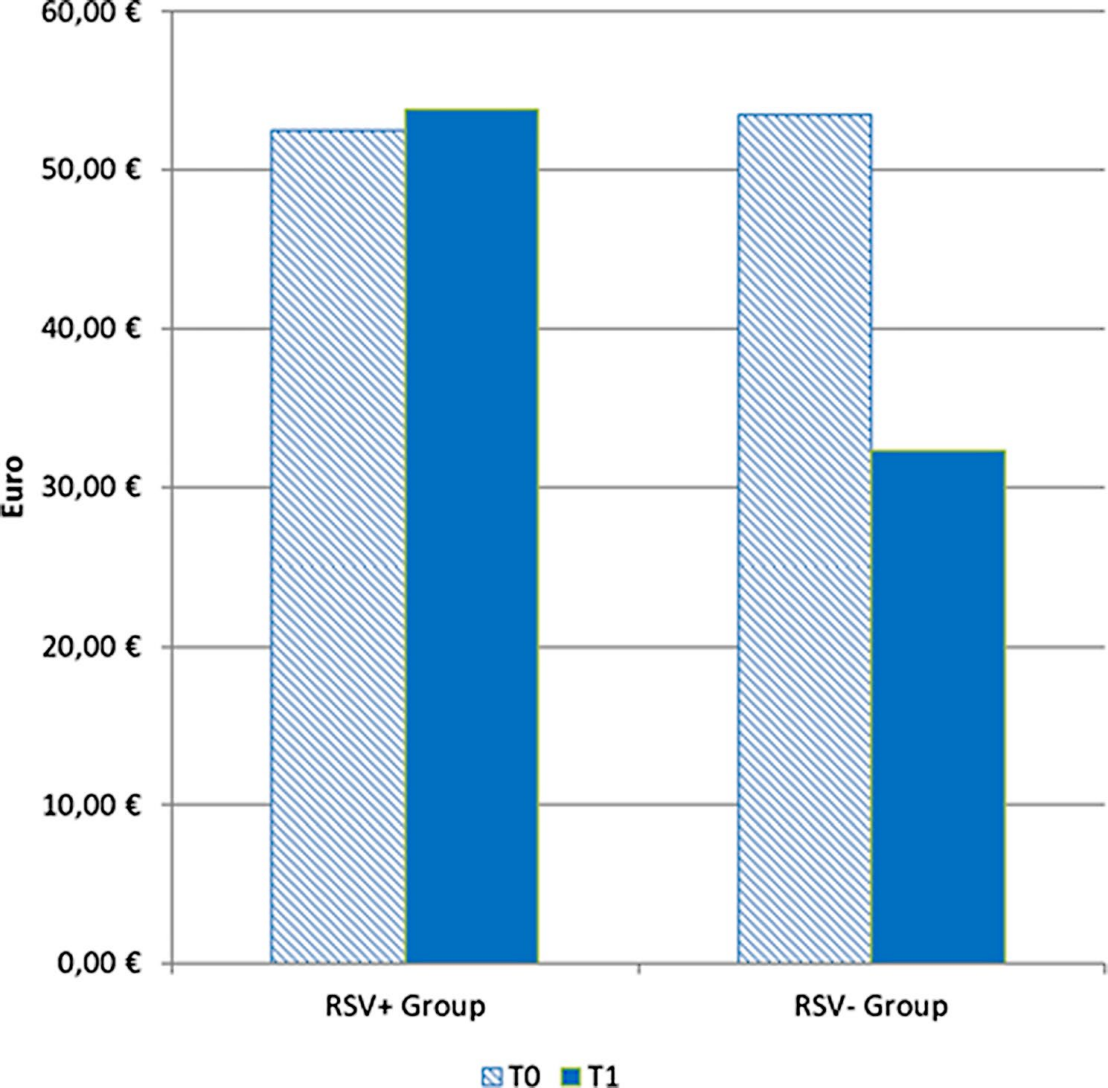
Direct costs paid by families for hospitalized patients

Additionally, the number of direct costs was similar at T0 (€ 52.5 ± 94.6 in RSV+ group and € 53.5 ± 171.4 in RSV- group), but higher at T1 in the RSV+ group (€ 53.8 ± 126.6 vs € 32.3 ± 64.4) (Fig. 1).

Regarding indirect costs, parents reported the need to take both paid and unpaid leave to care for their hospitalized children. At T1, 94 parents (41.8%) in the RSV-positive group missed work using paid vacation days, with a mean duration of absenteeism of 5.0 ± 2.9 days for one parent and 5.2 ± 3.2 days for the other. In contrast, 17 parents (25.35%) in the RSV-negative group reported taking paid leave, with a mean duration of 5.6 ± 2.1 days for one parent and 4.5 ± 2.5 days for the other. Comparison between the two groups indicated that RSV-positive parents required significantly more paid days off to care for their children (*p* = 0.0426).

In addition to paid leave, 31 parents (13.7%) in the RSV-positive group and 5 parents (7%) in the RSV-negative group reported taking unpaid leave. In the RSV-positive group, 12 parents took a mean of 4.5 ± 2.6 days and 19 parents took 3.6 ± 2.8 days. Among RSV-negative families, 3 parents required 6.0 ± 4.6 days and 2 parents required 7.5 ± 6.4 days of unpaid leave. Table 3 summarizes the results (Table 3).

**Table 3:**
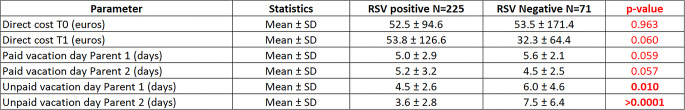
Direct and indirect costs for families of hospitalized patients

Based on the Italian standard monetary value of daily employee productivity, these absences translated into a total estimated income loss of €9,363.6 for RSV-positive families and €2,524.5 for RSV-negative families, illustrating that beyond the direct l costs of hospitalization, families potentially have to face significant indirect costs related to lost workdays, reduced income, and the need to reorganize dailylife.

## Discussion

Our study demonstrates the significant economic impact of RSV-related hospitalizations on families, capturing both direct out-of-pocket expenses and indirect costs due to work absenteeism across two consecutive epidemic seasons.

All Italian children receive free healthcare assistance from public hospitals, funded by taxes and national or provincial budgets. A small percentage of them have medical insurance that may cover extra-hospital expenses. In most cases, there are the so-called ‘‘out-of-pocket costs”, the medical and non-medical expenses paid out directly by the patient’s family. As a result, families are required to cover these costs and may have to reduce other expenses or use their savings. In fact, one family member permanently stays with the patient during hospitalization, since pediatric hospitals in the country usually allow only one caregiver per patient. As for the other parent, we have to consider the costs associated with transportation to visit the child at least once or twice for a hospital stay. Many people worldwide are vulnerable to the economic consequences of illness, regarding their socio-economic status. To face the cost, families may have to reduce other expenses or use their savings [14].

Supplemental childcare, over-the-counter medications, homemade remedies, and travel expenses resulted in an additional financial burden for families. Similar economic evaluations are scarce in literature and the inclusion criteria, as well as national healthcare policies, are different from each other, making comparison quite hard. In Europe, a study from Germany revealed medical costs of € 2,461 for each hospitalized case with ALRTI. In the report, the cost driver of direct medical costs for inpatient care was care on a regular pediatric ward and accounted for approximately 95% of direct medical costs. Parenteral expenses were responsible for 4% of the average total cost, with the extracted value being around € 98.44 [15]. In Spain, the median direct cost per RSV infant was of € 598.8 (IQR: 359.6–2,425.9). The lower mean cost may be linked to the inclusion criteria of both inpatients and outpatient children [16].

Regarding the indirect costs, a recent multicenter study, measuring indirect expenses due to RSV in a community setting in Europe, on infants less than 1 year old reported the median cost of all episodes being € 75.1 (IQR: 1.8–466.3) [17]. The most likely explanation for the observed difference in respect to our findings is dissimilarities in the design of the studies, but other potential reasons include differences in the relative severity of RSV seasons, younger populations studied, healthcare systems, and healthcare-seeking behavior.

**Fig. 2:**
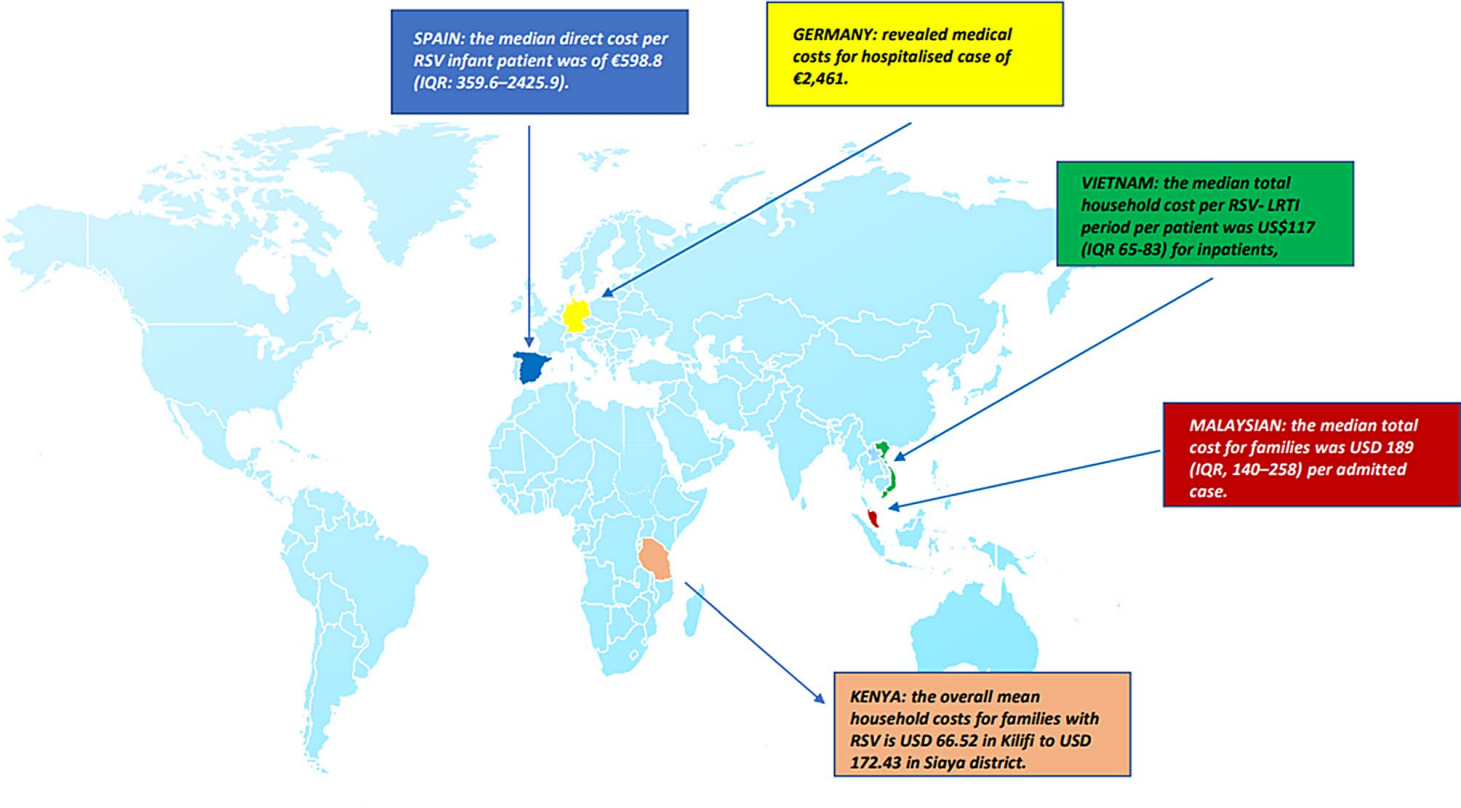
Overview of RSV direct and indirect costs in the world

In a Malaysian study, the median total direct family cost was 189 USD (IQR: 140–258) per admitted case [18]. This value is higher than our finding and may be explained by the fact that parents have to pay part of the hospital bills (median 135 USD [105–178]). Excluding the 65.6% of hospital costs, the family’s expenses are approximately 65.20 USD. In particular, the direct non-medical costs (comprising transportation to health facilities, parking, and petrol) amounted to 49 USD. Of note, the study population included children hospitalized with ALRTI by any etiological agent [18]. A previous data collection from 2015 to 2016 on RSV ALRTI hospitalized children reported cost to the household, which includes both direct and indirect costs incurred during the episode of illness, of 16.89 USD (IQR: 7.89–25.89), which roughly accounts for 20% of the total cost per episode of illness. Direct non-medical costs from the index visit, such as transportation and subsistence, account for approximately 38 and 45% of the total household cost among RSV-positive cases [19]. The difference in our findings may be explained by the different periods of study and the varying socio-economic status of the country. In Vietnam, the median total household cost per RSV- LRTI period per patient was 117 USD (IQR: 65–83) for inpatients, considering the expenses they incurred before, during, and after hospitalization. Parents of toddlers hospitalized for RSV incurred direct non-medical costs of 27 USD (IQR: 3–72), transportation costs of 8 USD (IQR: 2–41) and caretaker cost of 47 USD (IQR: 34–60). Indirect costs were estimated to be 18 USD (IQR: 0–69). They had also to participate in medical costs as are covered by the national health insurance system at 80% of the costs of listed medications and interventions [14]. In Kenya, the overall mean household costs for families with RSV- ALRTI hospitalized children ranged from 66.52 USD in Kilifi (95% CI: 53.50–79.55) to 172.43 USD (95% CI: 130.46 − 214.39) in Siaya district. The total mean cost incurred by a household for taking care of a child with RSV-associated LRTI during hospital stay was 46.88 USD (95% CI: 37.69–56.07) in Kilifi and 138.51 USD (95% CI: 106.54–170.48) in Siaya [20]. These data from extra-European countries are consistent with our findings, even though the settings are different. Figure 2 summarizes the evidence (Fig. 2).

The indirect cost of disease is an important factor and should be considered when measuring the additional impact of diseases beyond the direct costs. Indirect costs are costs of resources for which no payment is made, but which have a negative consequence for the family, such as missed days of vacation. Guidelines for economic evaluation studies in healthcare recommend the inclusion of indirect costs, though they are difficult to quantify due to a lack of quality data [21, 22]. The addition of indirect costs usually has a substantial effect on the efficiency ratio, especially in pediatric diseases, where hospitalization involves the entire family, increasing the economic impact of the disease on society. Excluding such costs also leads to an underestimate of the real cost of the disease and the effect that preventive interventions may have in reducing the frequency and duration of hospitalizations.

The number of workdays missed by parents differed significantly between the two groups, with RSV-positive families experiencing a greater burden. Specifically, a higher proportion of caregivers in the RSV+ group reported taking both paid and unpaid leave to care for their hospitalized child. This translated into a greater loss of productivity and, consequently, a higher indirect financial cost. The extended absence from work not only reflects the severity and demands of RSV-related hospitalizations but also highlights the broader socioeconomic implications for affected families. These findings are consistent with previous studies, which have similarly shown that RSV infections impose a disproportionately high indirect cost on families due to parental work absenteeism. The days of work absence of parents significantly differ among the two Groups, revealing additional indirect cost for RSV+ parents, in line with previous reports [18].

## Limitations and Strengths

A potential limitation of this study is the reliance on caregiver-reported expenses, which may introduce recall bias. Nevertheless, as parental out-of-pocket costs for hospitalized and nosocomial cases accounted for only a small fraction of the overall economic burden, the effect of such bias on total cost estimates is likely negligible. Another limitation is that we did not apply the human capital approach—the traditional method for estimating indirect costs through lost productivity—which may have led to a conservative assessment of the true socioeconomic impact of RSV-related hospitalizations. In addition, costs were standardized using questionnaires administered at admission and again after 7 days, rather than at hospital discharge.

Despite these limitations, the study has notable strengths. It was conducted prospectively across multiple centers representing different Italian regions, allowing for the collection of timely, high-quality data and ensuring a geographically diverse and representative sample of the pediatric population. This design enhances both the generalizability and the reliability of the findings.

## Conclusion

This study provides valuable insights into the economic burden of RSV-associated ALRTI hospitalizations on families, emphasizing both direct out-of-pocket expenses and indirect costs related to work absenteeism. By capturing the real-life impact of RSV on household activities and finances, our findings offer a crucial parent-centered perspective that supports a more comprehensive evaluation of RSV burden. These results can inform public health decision-making and guide policymakers in the adoption and prioritization of preventive strategies (such as maternal vaccination and monoclonal antibodies) to protect all infants during their first RSV season and those at continued risk during their second season. Future research should aim to explore long-term economic and psychosocial consequences of RSV infections on families, as well as assess the cost-effectiveness and equity impact of preventive interventions across different healthcare settings. Data on the impact of ALRTI among infants on the family activities support the evaluation of the burden of RSV from the parent perspective. Results from this study may guide recommendations for decision making towards implementation of RSV interventions by country policymakers and other global stakeholders to protect all infants at their first RSV season and those infants who remain vulnerable to severe RSV ALRTI through their second RSV season.

## Data Availability

Data and material are available at dr Bozzola’s office.

## Abbreviations

ALRTI: Acute lower respiratory tract infection
RSV: Respiratory syncytial virus
mAb: monoclonal antibody
INCIPIT: Italian Network for Paediatric Clinical Trials
